# Spatial and temporal patterns of sound production in East Greenland narwhals

**DOI:** 10.1371/journal.pone.0198295

**Published:** 2018-06-13

**Authors:** Susanna B. Blackwell, Outi M. Tervo, Alexander S. Conrad, Mikkel H. S. Sinding, Rikke G. Hansen, Susanne Ditlevsen, Mads Peter Heide-Jørgensen

**Affiliations:** 1 Greeneridge Sciences, Incorporated, Santa Barbara, California, United States of America; 2 Greenland Institute of Natural Resources, Copenhagen, Denmark; 3 Data Science Laboratory, Department of Mathematical Sciences, University of Copenhagen, Copenhagen, Denmark; Texas Christian University, UNITED STATES

## Abstract

Changes in climate are rapidly modifying the Arctic environment. As a result, human activities—and the sounds they produce—are predicted to increase in remote areas of Greenland, such as those inhabited by the narwhals (*Monodon monoceros*) of East Greenland. Meanwhile, nothing is known about these whales’ acoustic behavior or their reactions to anthropogenic sounds. This lack of knowledge was addressed by instrumenting six narwhals in Scoresby Sound (Aug 2013–2016) with Acousonde^™^ acoustic tags and satellite tags. Continuous recordings over up to seven days were used to describe the acoustic behavior of the whales, in particular their use of three types of sounds serving two different purposes: echolocation clicks and buzzes, which serve feeding, and calls, presumably used for social communication. Logistic regression models were used to assess the effects of location in time and space on buzzing and calling rates. Buzzes were mostly produced at depths of 350–650 m and buzzing rates were higher in one particular fjord, likely a preferred feeding area. Calls generally occurred at shallower depths (<100 m), with more than half of these calls occurring near the surface (<7 m), where the whales also spent more than half of their time. A period of silence following release, present in all subjects, was attributed to the capture and tagging operations, emphasizing the importance of longer (multi-day) records. This study provides basic life-history information on a poorly known species—and therefore control data in ongoing or future sound-effect studies.

## Introduction

Marine animals inhabiting high Arctic areas, such as the narwhal (*Monodon monoceros*), are seasonally exposed to extensive ice coverage with darkness prevailing in mid-winter and limited daylight for half of the year. Furthermore, narwhals dive extensively to depths much below the photic zone: in some areas they frequently dive to 1000 m and may occasionally dive below 1500 m [1, 2]. Considering their pack-ice habitat and bathypelagic foraging, narwhals therefore mostly depend on acoustics for sensing their environment, navigating the underwater icescape, capturing prey at depth, and communicating with conspecifics.

Early studies of narwhal sounds [3–6] described the fundamental components of their vocal repertoire, *i*.*e*., clicks, burst pulses, and whistles. Technological improvements, such as the ability to record at higher sampling rates, later led to more accurate or complete descriptions of these sounds [7, 8]. A few other studies have investigated behavioral aspects of the vocal repertoire, such as the possible use of “signature” calls [9], the vocal repertoire during the winter [10], and the possible relationship between call use and behavioral state [11]. All these studies were based on data collected in Canada and West Greenland, whereas the data presented here are, to our knowledge, the first acoustic records acquired from East Greenland narwhals. Narwhals from the east and west sides of Greenland have been separated at least since the end of the last glaciation, more than 10,000 years ago, enough time to have led to genetic differentiation [12].

In most of the acoustic studies mentioned above, data were collected with dipping hydrophones, autonomous passive acoustic recorders, or hydrophone arrays. Such studies are generally limited to describing the acoustic signals received at the recorders, with little information on the spatial and temporal variation in sound production of specific individuals. The development of animal-borne acoustic recorders has opened up new options for monitoring the individual acoustic behavior of free-ranging cetaceans [13]. High-resolution acoustic recorders have traditionally been attached to the skin of whales with suction cups. As a result, attachment duration has usually been short, often less than 24 h [14], including two deployments of less than 3 h and 13 h on narwhals in Canada [9]. Short-term deployments provide little information on diel patterns or variability over time for whales that are frequenting different habitats. Furthermore, there is a risk that the data from short-term deployments are affected by the whales’ reaction to tagging, meaning they are not representative of the animal’s normal behavior.

Narwhals are known to live in pristine environments with limited human activities, of which hunting may have the greatest impact (https://nammco.no/topics/narwhal/). The increase in average temperatures in the Arctic and the concomitant loss of summer sea ice could change the state of these pristine environments [15]. Increases in ship traffic and exploration for minerals have rendered narwhal habitats more exposed to noise pollution. Of imminent concern is seismic exploration for oil and gas, which could lead to disturbances of migratory corridors and summering grounds [16], as well as to behavioral effects such as changes in calling or feeding behavior—all of which could lead to population consequences for the whales.

The ability to detect such changes is dependent on knowledge of the whales’ normal behavior, gathered in the absence of industrial sounds. Collecting this knowledge is one of the goals of this study. The narwhals that summer in Scoresby Sound are not completely naïve to anthropogenic sounds. In mid-winter, airgun pulses have been recorded in Fram Strait [17], north of where the Scoresby Sound population is known to overwinter [2]. (The origin of the airgun pulses mentioned in [17] was unknown, but is likely in more temperate, ice-free waters.) Furthermore, there is a single village of <500 inhabitants within the fjord complex, and therefore associated low levels of vessel traffic in the summer. Nevertheless, the overall received levels of anthropogenic sounds experienced by these whales—at least until recently—are likely some of the lowest to be found in the marine environment.

Narwhals are skittish and cannot be approached at sea for tagging. Instead, it is necessary to live-capture the whales in nets in order to instrument them. Such operations can only be conducted in certain locations, where narwhals are known to pass during their migrations and where field facilities, including accommodation and boats, can be maintained. Additionally, to study the vocal behavior beyond the first day after tagging, when the whale may be in a different part of its range and performing different behaviors, long-term deployments are necessary.

Considering the scarcity of information on the effects of underwater sound on narwhals (*e*.*g*., [18]) and the complete lack of acoustic studies on East Greenland narwhals, it seems imperative to gather such information while anthropogenic disturbances are still at a relatively low level. This study therefore aimed to address some of these gaps in knowledge. Specifically, we aimed to (1) gather information on the vocalizations of East Greenland narwhals in a coastal summering ground (Scoresby Sound), over the widest temporal and spatial scales possible, (2) relate the use of clicks, buzzes, and calls to the spatial and temporal utilization of the summering ground, and (3) quantify the immediate effects of handling and tagging on the whales’ acoustic behavior. In addition to the intrinsic value of having such basic life-history information, establishing these baselines is particularly important for assessing the effects of future anthropogenic sounds, such as airguns or vessels, on narwhal behavior.

## Materials and methods

### Field tagging

**Fig 1:**
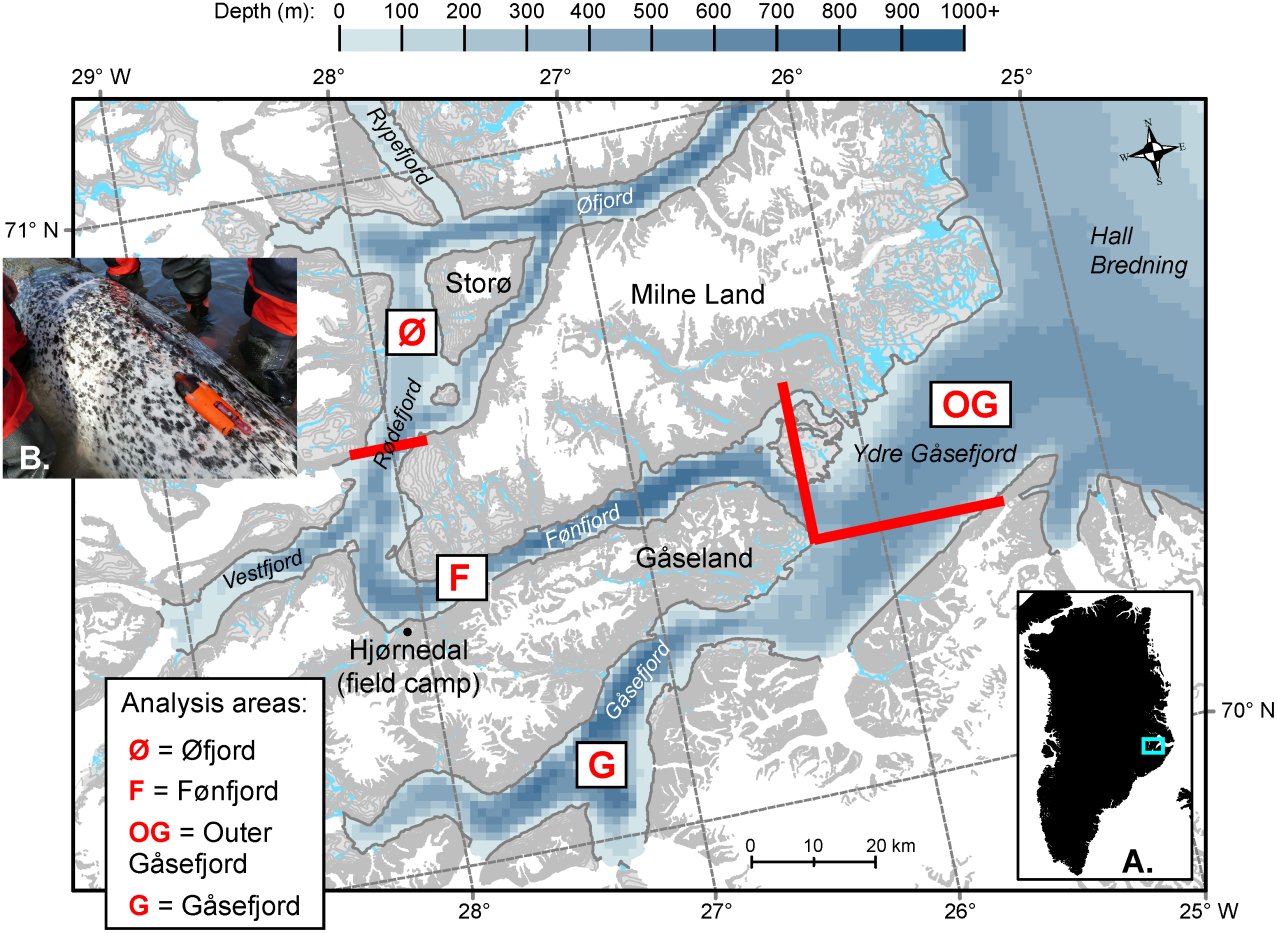
Detail of study area in Scoresby Sound, East Greenland, as shown in (A) with the turquoise rectangle. The four sub-areas used in the statistical analyses are delimited by the thick red lines: Øfjord (Ø) to the northwest; Fønfjord (F), which includes the tagging area and the fjord along the southwestern shore of Milne Land; Gåsefjord (G), the large fjord south of Gåseland; and Outer Gåsefjord (OG), where Gåsefjord broadens and merges into Hall Bredning, at the widest part of Scoresby Sound. (B) Acousonde on female Freya, 8 Aug. 2013 (picture by MP Heide-Jørgensen). The map was generated with ArcGIS using bathymetric data from the International Bathymetric Chart of the Arctic Ocean (IBCAO) and coastline data from the Geological Survey of Denmark and Greenland (GEUS) [19].

Narwhals were live-captured in August 2013–2016 from a field station (Hjørnedal) near the southwestern tip of Milne Land in the Scoresby Sound fjord complex (Fig 1). Whale captures were accomplished using set nets (40 or 80 m length, 5–8 m deep) in collaboration with local Inuit hunters (see [2] for more information). Handling of captured whales was conducted near shore by six persons in survival suits standing on either side of the whale to restrict its movements and support it, if needed. Gender of the whale was determined based on presence (male) or absence (female) of a tusk.

Five female narwhals, one accompanied by a calf, and one male narwhal (Table 1) were instrumented with Acousonde^™^ acoustic and orientation tags (www.acousonde.com), whose float had been modified to accommodate an Argos transmitter (Wildlife Computers SPOT5) in addition to a VHF transmitter (ATS Telemetry). The Acousonde recorders were attached to the skin with suction cups, on the rear half of the animal, to the side of the dorsal ridge (Fig 1B). To extend the longevity of the attachment, one or two 1-mm nylon lines were threaded through the top of the dorsal ridge. The Acousonde was held to the lines with magnesium corrodible links, which ensured release of the recorder after 3–8 days of attachment (Table 1). Once detached from the whale, the Argos transmitter provided its position to the field station, and the VHS transmitter enabled close-up relocation of the package. All deployed tags were retrieved 1–4 days after their release from the whale.

**Table 1:** Morphometrics and record information for instrumented whales. Body mass is estimated from standard length [20]. Based on his length, Balder is considered immature. The females are considered adult, but Thora only marginally so.

Whale (sex)	Length (cm)	Fluke width (cm)	Mass (kg)	Year	Record start	Record end	Record duration hh:mm (d)
Freya (F)	420	93	1045	2013	8 Aug. 16:41	11 Aug. 12:55	68:14 (2.8)
Thora (F)	341	84	560	2014	11 Aug. 15:32	15 Aug. 22:19	102:47 (4.3)
Mára (F)	390	95	824	2014	11 Aug. 16:15	12 Aug. 02:15	10:00 (0.4)
Frida (F)	380	85	762	2015	15 Aug. 18:31	19 Aug. 05:12	82:41 (3.4)
Eistla (F) ^a^	~360 ^c^	-	650	2016	24 Aug. 13:16	28 Aug. 19:47	102:31 (4.3)
Balder (M) ^b^	372	90	685	2016	24 Aug. 12:50	31 Aug. 11:54	167:04 (7.0)
SUM:							533:17 (22.2)

^a^ Accompanied by a calf

^b^ Tusk length 74 cm

^c^ Estimated

In addition to the Acousonde, a satellite transmitter was also deployed on the whales for long-term tracking following procedures described in Heide-Jørgensen *et al*. [2, 21]. The females were instrumented with tags from Wildlife Computers (Redmond, WA; Freya: Mk10; Thora, Mára, and Frida: SPLASH tag; Eistla: SPLASH tag with Fastloc^®^ GPS option), while Balder, the male, was instrumented with a CTD tag from SMRU (Sea Mammal Research Unit, St Andrews, UK). Eistla’s calf remained nearby while its mother was being instrumented. Both mother and calf were observed leaving together after Eistla’s release.

### Permitting

Permission for capturing, handling, and tagging of narwhals was provided by the Government of Greenland (Case ID 2010–035453, document number 429 926). The project was reviewed and approved by the IACUC of the University of Copenhagen (17 June 2015). Access and permits to use land facilities in Scoresby Sound were provided by the Government of Greenland. No protected species were sampled.

### Sampling regimens

Of the Acousonde’s eleven recording channels, three are relevant to the analyses presented in this paper: the two acoustic sampling channels and the depth measurements. The “low-frequency” (LF) acoustic channel included an HTI-96-MIN hydrophone with a nominal sensitivity of -201 dB re 1 V / μPa, a preamp gain of 14 dB, and an anti-aliasing filter with a 3-dB reduction at 9.2 kHz and a 22-dB reduction at 11.1 kHz. The “high-frequency” (HF) acoustic channel included an HTI-99-HF hydrophone with a nominal sensitivity of -204 dB re 1 V / μPa, a preamp gain of 29 dB, and an anti-aliasing filter with a 3-dB reduction at 42 kHz, and a 22-dB reduction at 100 kHz. All acoustic data were recorded with 16-bit resolution (for further technical information about the Acousonde, visit www.acousonde.com).

Two different acoustic sampling regimens were used, both providing continuous acoustic records. The first, used on Freya, Thora, Mára, and Eistla (Table 1), alternated sampling on the two hydrophones: 8 min at a sampling rate of 25,811 Hz on the LF channel followed by 7 min at 154,868 Hz on the HF channel. The second sampling regimen, used on Frida and Balder, sampled continuously on the LF channel at a 25,811 Hz sampling rate. These choices were determined by storage and battery considerations—with alternating LF / HF sampling the tag filled to capacity in ~4.2 days; when sampling continuously at low frequency, acoustic recording ended after ~7 days due to low battery voltage.

Depth was sampled at 10 Hz in all subjects. After retrieval, all depth records were averaged to a 1 Hz sampling rate and, when necessary, the surface value was reset to a depth of 0 m.

### Sound analyses

The analyses focused on three types of sound—clicks, buzzes, and calls. Clicks and buzzes are generally associated with feeding by echolocation, but can also be used for general orientation in the environment (*e*.*g*., [22, 23, 24] for a review). The “call” category, as used in this study, includes whistles, pulsed calls, and other vocalizations. This broad category is generally thought to serve communication between conspecifics [4].

#### Clicks

All sound files (n = 3135) were examined manually as a sound pressure time series (SPTS) by two analysts using MTViewer (a custom-written program for analysis of Acousonde data, W.C. Burgess, pers. com.). To reduce the contribution of flow noise and make the times of clicking obvious in the SPTS, files from the LF channel were high-pass filtered at 1.5 kHz (see examples in supporting information S1 Fig and S1 File with S5 and S6 Audio). HF files did not require any filtering to reveal the sounds of interest (see S1 Fig and S1 File with S1, S2, S3 and S4 Audio), as the Acousonde has a built-in high-pass filter cut-off frequency of 9.85 kHz. SPTS of the acoustic recordings were examined at standardized x- and y-axis resolutions, and frequent checks for consistency between analysts were performed. During dives, the times at which the whales started clicking during descent and stopped clicking during ascent were noted. Brief interruptions in clicking at depth, generally lasting a few s (S1 Fig) and rarely up to 10 s or more, were ignored. Some whales also clicked at or near the surface, where clicking bouts were short, often lasting less than 30 s. In these cases interruptions in clicking of more than 10 s defined a new clicking bout.

The source of the clicks—tag-bearing whale or other narwhals—was mainly determined using aural qualities, combined with visual cues. Differences in the length and frequency composition of clicks produced by the tagged whale versus other (non-tagged) whales have been described by Zimmer *et al*. [25], Johnson *et al*. [13], and Johnson [26]. Basically, due to the tag being connected to the whale’s body through the suction cups, clicks produced by the tag-bearer have a low-frequency component (see [27] and supporting information S2 Fig) that reaches the tag through tissue conduction. This low-frequency component is usually obvious when listening to the sound records (see S1 File and S1, S2 and S3 Audio). Clicks produced by the tag-bearer are also much longer in duration [26]. In addition, click trains produced by the tag-bearer include smooth changes in amplitude between successive clicks, as shown in S1 Fig. Click trains from other (non-tagged) narwhals on the Acousonde record were generally short (a few seconds) and often included rapid changes in amplitude between successive clicks. Both of these factors can be explained by the random movements of whales with respect to the tagged whale, combined with the highly directional echolocation beam of narwhals [8].

#### Buzzes

A custom-written buzz detector (Matlab, The MathWorks, Inc., Natick, MA, USA) was used to identify buzzes in the records. The detector looked for periods of sustained energy that were temporally associated with clicks and occurred at certain frequencies. The detector was adjusted to miss a minimal number of buzzes, but as a result it produced a fair number of false detections. All buzzes output by the detector were therefore checked by a manual analyst. To allow evaluation of the detector’s performance, a subset of sound files (~30%) were also analyzed manually for the presence of buzzes. Examples of buzzes are shown in S1 Fig and also S1 File with S4, S5 and S6 Audio.

The buzz detector did not provide reliable buzz durations. A subset of buzzes was therefore analyzed manually: 35 buzzes per whale, selected with a random number generator, and 18 for Mára (all of the buzzes included in her short record). Unlike what is observed in beaked whales [28], there is in narwhals a progressive transition between regular clicking and buzzing. Inter-click intervals (ICIs), which are generally in the range 80–250 ms during regular clicking, decrease progressively to 2–8 ms during the buzz itself. The transition is generally rapid, lasting less than 0.5 s, but occasionally lasts for several seconds. For the purpose of this analysis, we therefore defined the buzz as beginning when the ICI decreased below 50 ms.

#### Calls

During manual review of the sound files for periods of clicking (see above), the presence of calls produced by the tag-bearing whale or other nearby whales was noted. With “call” we mean any vocalizations that were not regular clicks and buzzes. These sounds were mainly comprised of click-based calls such as burst pulses, but also included other sounds such as whistles. As it is beyond the scope of this paper to analyze these calls in detail, they were lumped into a single category, the function of which we assume is primarily social communication.

Click-based calls such as burst pulses were distinguished from the terminal buzzes used during feeding (see above) by the fact that they were not preceded or followed by regular echolocation clicks. In addition, calls were generally high-amplitude sounds (S1 File and S7 Audio), contrary to buzzes, during which the whales decrease both the inter-click interval and the amplitude of their clicks (S1 Fig and S1 File with S3 and S5 Audio; see also [24] for a review).

We did not attempt to limit our analysis to calls produced by the tag-bearing whale, for the following reasons. First, the source—tag-bearer or nearby narwhal—of tonal sounds such as whistles cannot be determined using frequency characteristics recorded on either acoustic channel, as explained above for clicks. In addition, even though low-amplitude sounds, such as buzzes, are only picked up by the Acousonde on the LF channel if they are produced by the tag-bearer, this is not true of high-amplitude sounds, such as burst pulses. The source of these sounds can therefore be difficult to ascribe based on the LF recordings, particularly in social situations involving two or more narwhals in close proximity (S1 File and S7 Audio). For these reasons, we deemed it better to include all calls detected and use them as a relative indicator of ongoing communication between the whales.

### Combining Acousonde data with satellite track information

An important question for us was to investigate how buzzing and calling rates varied as a function of depth, time, and the whales’ geographic location within Scoresby Sound. To this effect we split our study area into four coarse subareas, shown in Fig 1: Øfjord (Ø, north of the field camp, along the north shore of Milne Land), Fønfjord (F, fjord along the southern shore of Milne Land), Gåsefjord (G, large fjord south of Gåseland), and the eastern Outer Gåsefjord (OG). We then used the nearest Argos / GPS position from the whales’ satellite tags to assign each second of the whales’ tracks to the different subareas.

A more detailed visualization of buzzing and calling rates was possible for the two females Thora and Eistla, because their positional information was dense enough that we could assign buzzes and calls to specific locations of their tracks. Thora had one long gap, of 5.6 h, between successive positions, with a change in location of more than 16 km. For this one gap we added an intermediary position midway—in both space and time—between the two positions framing the gap. Each buzz or call in Thora’s and Eistla’s records was allocated to the nearest Argos or GPS position. Buzzing and calling activity were then expressed for each track position in buzzes vs calls / min. The median distance separating a buzz or call and its assigned position was 2043 m for buzzes and 891 m for calls. These values were calculated using the median time separation between a buzz or call and its assigned position for Thora’s and Eistla’s combined records, and an average travelling speed of 1.5 m / s.

### Statistical analyses

Logistic regression models were used to assess the effects of location in time and space on buzzing and calling rates. The presence or absence of a buzz or call in each second of the datasets was used as the response, while the explanatory variables were *depth* (non-linearly with natural splines with 3 degrees of freedom, except for one case [Balder] with 2 degrees of freedom to ensure convergence) derived from the record, *time-of-day* (with periodic B-splines with 3 degrees of freedom and boundary knots at 0 and 24 h) and *subarea*. Datasets from each whale were analyzed separately, but Mára was left out of these analyses as her record only included 1 h of clicking and buzzing. Once Freya started echolocating she only buzzed and called in one subarea (G), so the effect of *subarea* on buzz or call production could not be evaluated. For the other whales, the effect of *subarea* was only assessed for the subareas visited by each whale. The procedure glm with logit link function in R (version 3.4.1; [29]) were used for the analyses. For the natural splines, the procedure ns from the package splines (base package) were used, and for the periodic splines, the procedure pbs from the package pbs (version 1.1) was used.

## Results

Over 533 h (22.2 days) of on-whale acoustic recordings were obtained from all animals combined (Table 1). Thora, Eistla, and Balder’s tags detached after filling to capacity. Freya and Frida’s records are shorter because the tag detached from the whale before recording ended; Mára’s record ended prematurely due to a power reset. Therefore, Mára’s data are excluded from many of the analyses.

**Fig 2:**
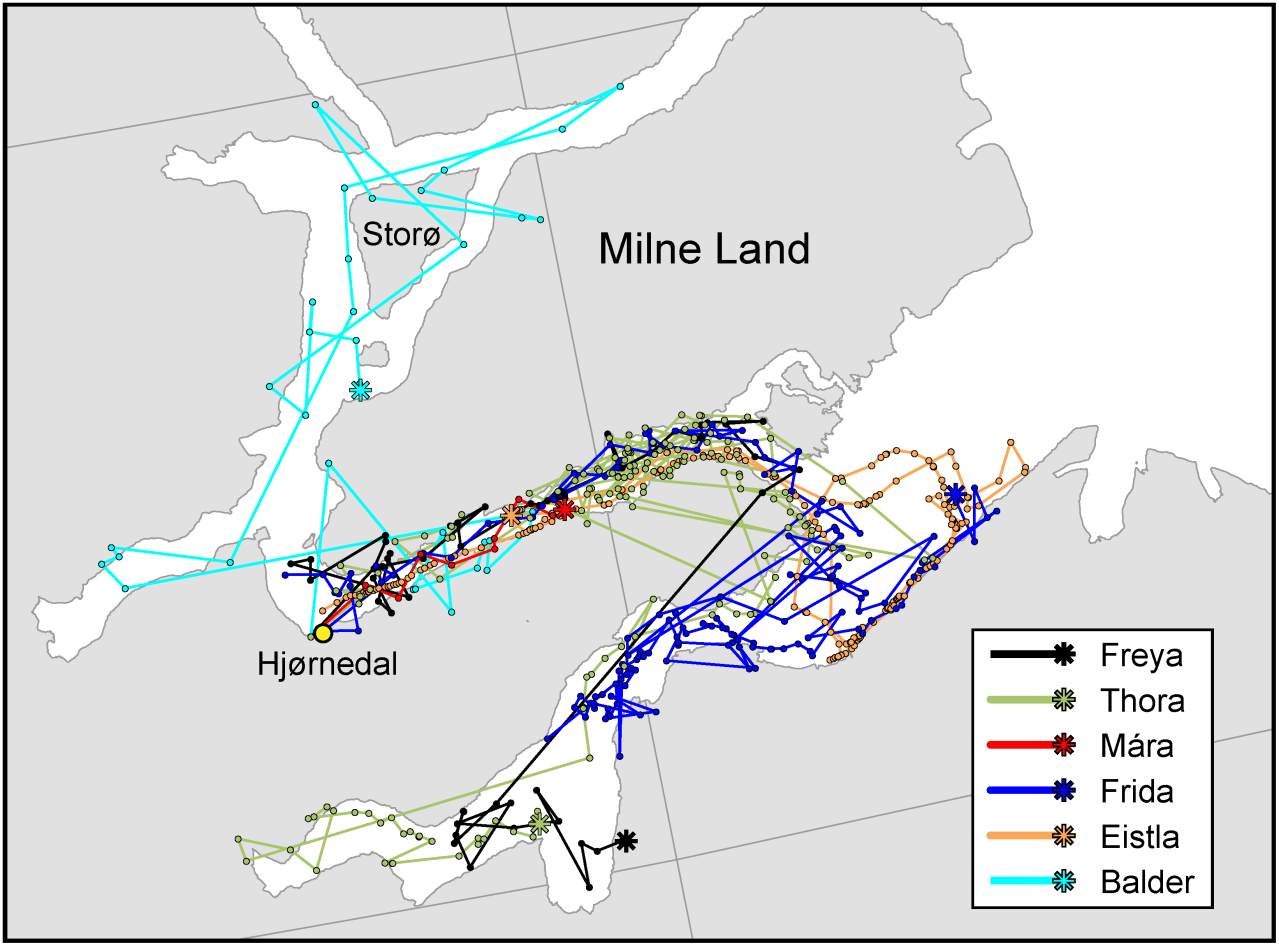
Satellite tracks of the six subjects over the duration of their Acousonde records. All tracks begin near the field camp (Hjørnedal), shown with a yellow dot, and end at the star symbol for each whale. The map was generated with ArcGIS using coastline data from the Geological Survey of Denmark and Greenland (GEUS) [19].

All whales departed from the area where they were captured (Hjørnedal) right after their release and headed east into Fønfjord (area F), south of Milne Land (Fig 2). Most of the females then went on to Gåsefjord and / or Outer Gåsefjord (areas G and OG), whereas the male reversed direction, came around the southwestern tip of Milne Land and headed northeast into Øfjord, towards Storø, west of Milne Land (area Ø). Tracks of all animals are summarized in Fig 2.

### Clicking behavior

**Fig 3:**
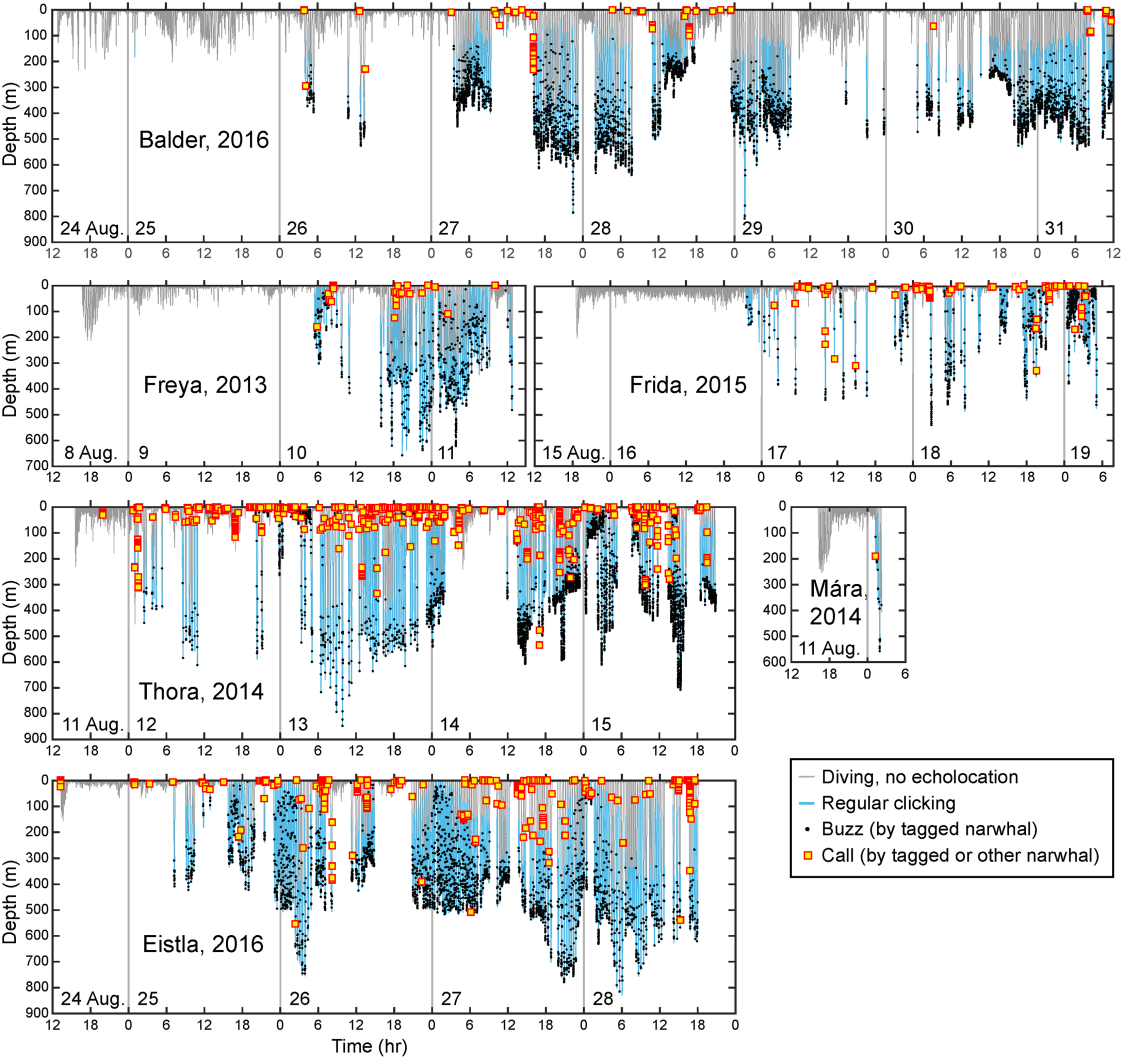
Dive depth as a function of time (gray lines) for all instrumented whales in 2013–2016. Periods of echolocation by the tagged whale are shown in blue and each buzz is indicated by a black dot. The red-edged yellow squares show calls made either by the whale carrying the tag or other whales nearby (see text for more information).

None of the whales started echolocating immediately following release. Instead, they remained silent for an average of 23 h (range 9–37 h, n = 6, Table 2, Fig 3) before clicking and buzzing began. Balder clicked for less than 30 s about 12 h after release, then remained silent for another 24 h before clicking and buzzing began in earnest. Once echolocation began, whales spent on average 27% of their time clicking (range 18–37%, n = 5, Table 2).

**Table 2:** Various parameters related to clicking behavior (echolocation). The percentage of time spent clicking excludes the post-release silent period. Clicking bouts of less than 1 min in duration are excluded from the five rightmost columns of data.

Whale	Start of clicking	Length of post-release silence (h)	% time spent clicking	Clicking bouts				Mean depth (S.D.), as % of max depth	
				n (>1 min)	Mean (max) duration (mm:ss)	Mean (S.D.) depth (m)	Max depth (m)	At start of clicking	At end of clicking
Freya	10 Aug. 05:28	36.8	32.7%	75 (74)	8:21 (13:11)	267.7 (146.0)	661	11.8 (15.1)	62.1 (16.0)
Thora	12 Aug. 00:58	9.4	27.6%	387 (220)	6:42 (16:04)	286.3 (185.6)	848	12.3 (14.5)	53.5 (25.8)
Mára	12 Aug. 01:14	9.0	42.7% ^b^	5 (5)	5:10 (8:29)	298.5 ^b^ (139.9)	563	20.1 (7.6)	94.2 (7.3)
Frida	16 Aug. 21:30	27.0	18.8%	192 (107)	5:36 (15:58)	128.0 (124.5)	540	6.7 (12.9)	37.4 (34.5)
Eistla	25 Aug. 07:10	17.9	36.6%	271 (175)	10:08 (15:08)	359.9 (169.1)	829	17.8 (20.0)	49.6 (19.1)
Balder	26 Aug. 00:55	36.1 ^a^	18.2%	204 (188)	7:34 (12:18)	356.6 (121.4)	810	32.8 (15.6)	75.1 (12.1)
MEAN:		22.7	26.8%			280 m			

^a^ This duration ignores 23 s of clicking that took place 12.2 h after release

^b^ Not used in mean at bottom of table because clicking began only 1 h before end of record

**Fig 4:**
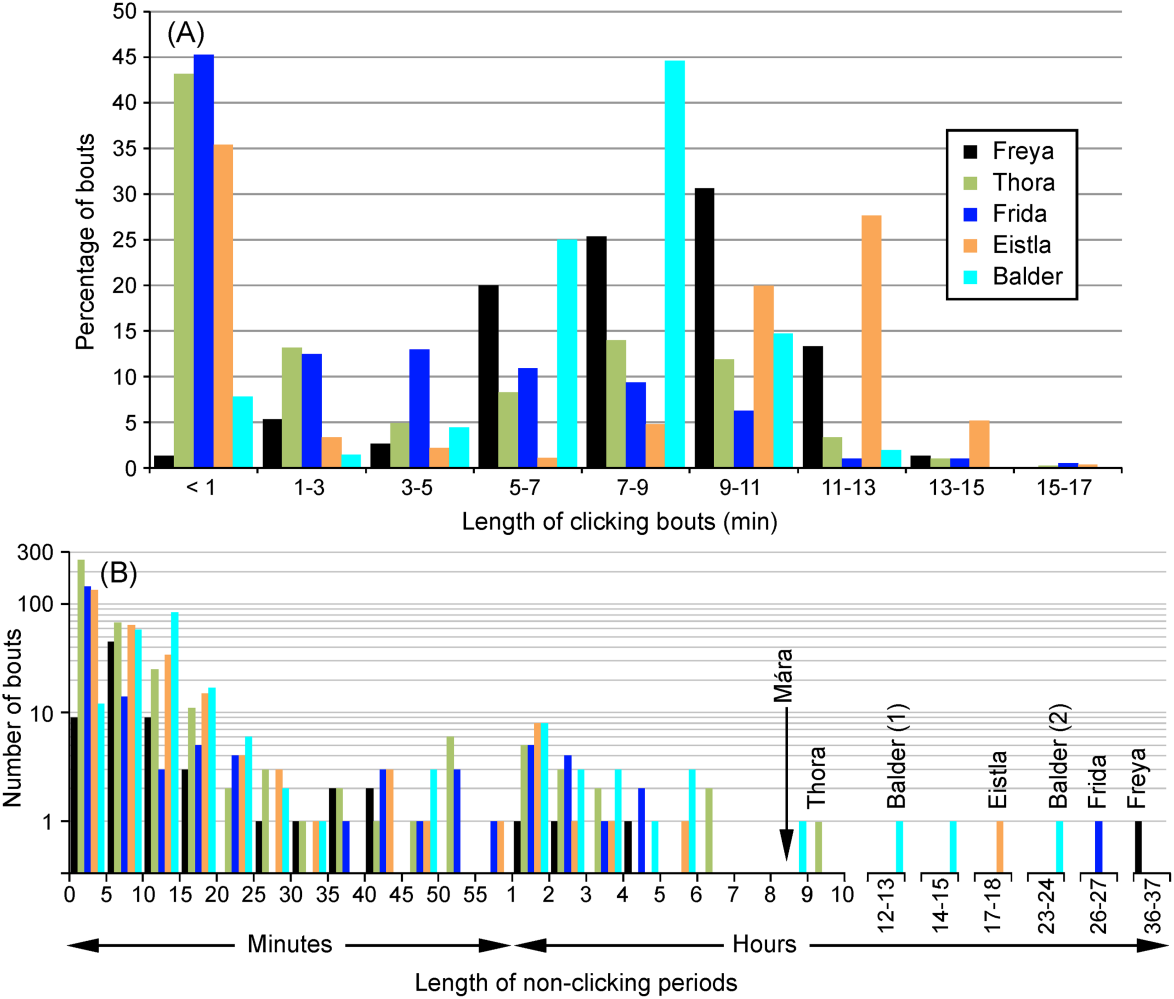
Histograms of the length of clicking and non-clicking periods, for all records but Mára’s. (A) Percentage distribution of clicking bouts. Each bin on the x-axis is 2 min wide except for the first one (<1 min). (B) Number of click-free bouts of various durations. The y-axis above 1 is logarithmic because of the range of values. Bins on the x-axis have a width of 5 min from 0–60 min, then a width of 1 hour from 1–37 h. Bins above 10 h are non-continuous and are only shown if they contained data. The whales’ names are placed above the first click-free bout of each record, following release. Two bouts [(1) and (2)] are shown for Balder, since he used echolocation for 23 s about 12 h after release, following which he was silent for another 24 h. The vertical arrow shows the length of the non-clicking period following Mára’s release (her data were not otherwise included in these plots).

Mean depth during clicking was 280 m but varied between whales (range 128–360 m). Three of the females, Thora, Frida, and Eistla, had a high percentage (35–45%) of clicking bouts lasting less than one min (Fig 4A). These bouts generally took place near the surface: for example, 90% of Thora’s clicking bouts that lasted less than one min were at depths of less than 10 m. Corresponding values for Frida and Eistla were 95% and 68%, respectively. These short clicking bouts were excluded in the calculation of mean clicking bout duration, which ranged from ~5.5 to 10 min (Table 2). Maximum clicking bout duration was 12.2–16 min (Table 2, excluding Mára). The whales generally began clicking at a shallower depth on descent than that at which they stopped clicking on ascent (Table 2).

For Thora, Frida, and Eistla, the most common echolocation-free period was less than 5 min long (Fig 4B), matching their frequent clicking behavior at or near the surface. Echolocation-free periods for Freya and Balder, who clicked much less at the surface (Fig 4A), were most commonly 5–10 min and 10–15 min long, respectively, between clicking bouts. The initial click-free period that followed release was the longest such period for any of the whales (Fig 4B). Balder’s long record did include one other 14.1 h period devoid of clicking, in the early part of his record (26–27 Aug., as shown in Fig 3).

### Buzzes: Numbers, depth distribution, and buzz durations

The buzz detector identified a total of 16,970 buzzes from all files on which it was used, of which 78% (13,236) were correct and 22% were false detections. The detector’s performance was assessed manually using 962 files (both LF and HF) from the records of Freya, Thora, Eistla, and Balder. Overall, the buzz detector identified about 95% of the buzzes detected during manual analysis: 91% for Freya, 95% for Thora, 97% for Eistla, and 98% for Balder. Differences between whales were in large part related to the signal-to-noise ratio (SNR) of files. Manual analysis, used here as the golden standard, was not perfect: in the LF files, which generally had a lower SNR than the HF files, the detector sometimes identified buzzes that were missed by human analysts.

Mean buzzing rate, calculated from the onset of clicking until the end of each record, ranged between 16 and 47 buzzes per hour, while maximum buzzing rate varied between 72 and 250 buzzes / h for the longer records (Table 3). At least three buzzing “styles” were observed: buzzes concentrated at the deepest depths of dives (*e*.*g*., Thora and Balder, Fig 3), buzzes near the surface (*e*.*g*., Thora and Frida), and buzzes that are loosely scattered throughout the dives (*e*.*g*., Freya, and parts of Thora and Eistla’s records).

**Table 3:** Various parameters related to buzzes and calls. Buzzes only include those made by the tagged whale, whereas calls include those made by the tagged whale and other nearby whales. The calculation of mean buzzing and calling rates omitted the post-release silent period. A few calls in Thora’s and Eistla’s records, which took place before the tagged narwhal started clicking, were excluded (see Fig 3). IQR = inter-quartile range, NA = not applicable. All depths are in meters. Buzz durations are based on 35 analyzed buzzes per whale, and 18 for Mára.

Whale	B U Z Z E S					C A L L S		
	n	Buzzes / h		Depth	Duration	n	Calls / h	Depth
		Mean	Max	Mean ± SD (min-max)	Median (s) (IQR)			Mean ± SD (median)
Freya	645	20.5	72	319 ± 148 (14–657)	2.5 (1.7–3.2)	39	1.2	25.1 ± 38.1 (6.7)
Thora	4301	47.0	250	325 ± 175 (2–847)	1.5 (1.1–1.9)	768	8.3	39.7 ± 70.8 (5.5)
Mára	18	18.1	18	361 ± 144 (115–557)	2.3 (1.6–3.2)	1	NA	NA
Frida	887	15.9	119	171 ± 135 (0–540)	2.7 (1.9–3.5)	149	2.7	29.2 ± 57.1 (12.5)
Eistla	2798	33.7	89	391 ± 151 (5–816)	2.7 (1.7–4.1)	530	6.1	30.8 ± 73.6 (1.3)
Balder	4587	36.0	175	374 ± 107 (96–808)	2.7 (2.3–3.7)	148	1.1	34.4 ± 63.1 (2.0)

For the three whales with alternating LF / HF sampling and multi-day records—Freya, Thora, and Eistla—the ratio of buzzes detected during LF vs HF sampling was used to ascertain that the detector performed equally well on both types of acoustic data. LF sampling occurred 53.5% of the time (8 out of 15 min) and the percentage of buzzes detected during LF sampling was close to expected for all three females: 54.0% for Freya, 53.3% for Thora, and 51.8% for Eistla.

**Fig 5:**
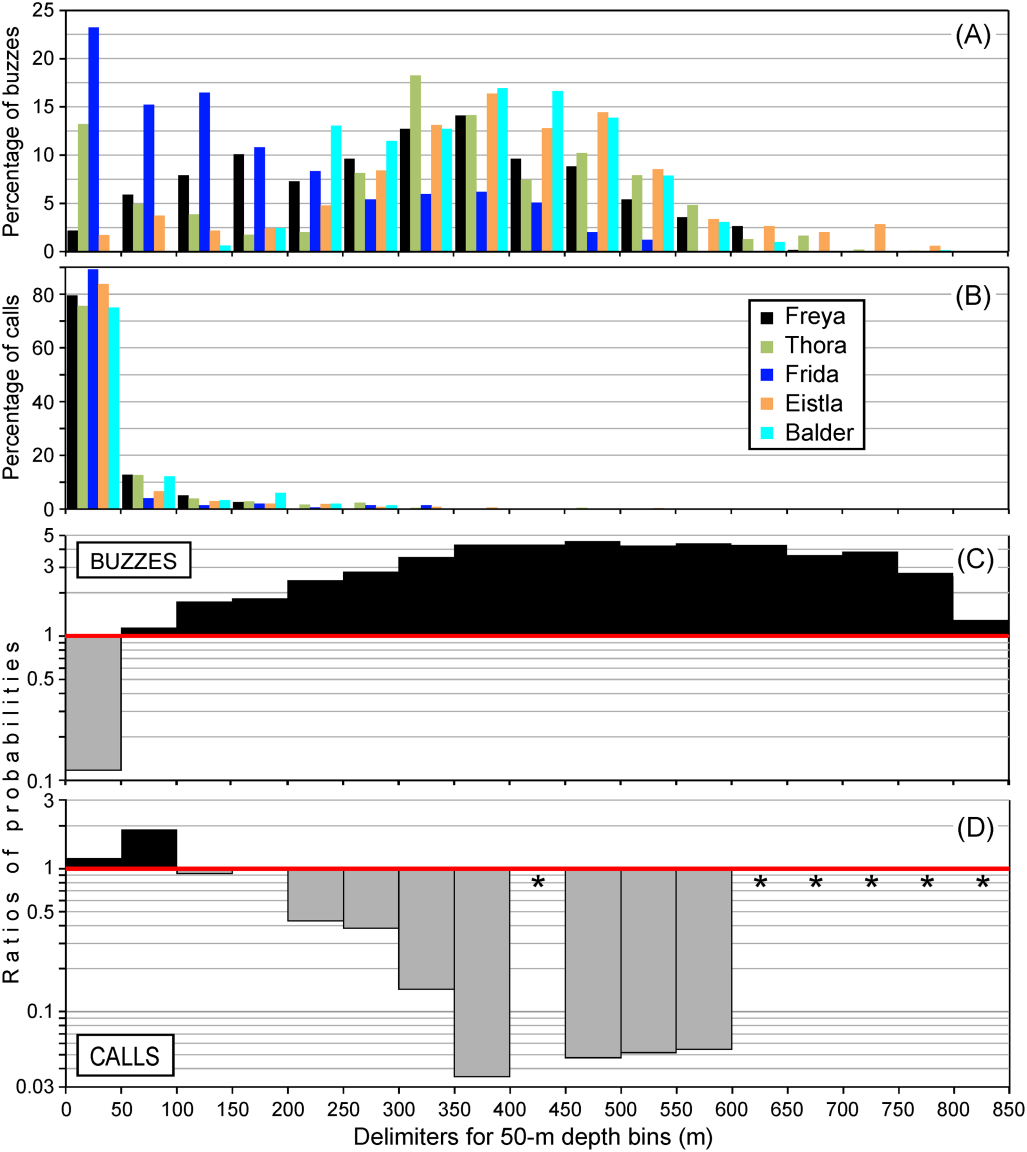
Preferential depths for buzz and call production, for 17 50-m depth bins encompassing the range of depths visited by the whales (all but Mára). (A) Depth distribution of buzzes produced by the tag-bearer. (B) Depth distribution of calls detected in the acoustic records. All calls detected were included, *i*.*e*., calls made by the tag-bearer as well as other nearby whales. (C) and (D) show ratios of probabilities for (C) buzzes and (D) calls, for the combined records. (See text for more information.) Ratios >1, shown in black, indicate a higher-than-expected rate of buzzing or calling, while ratios <1, shown in gray, indicate a lower-than-expected rate. The star symbols indicate depth bins that did not include any calls, so for which ratios of probabilities could not be calculated.

Mean buzz depth was in the range 319–391 m, except for Frida for which it was much shallower (~170 m, Table 3). The 50-m bin (Fig 5A) with the peak number of buzzes was between 300 m and 400 m for all whales but Frida, who displayed a different buzzing pattern: nearly 55% of her buzzes were produced in the top 150 m, compared to Balder, for example, who produced less than 1% of his buzzes in the top 150 m.

For each whale, we also computed the percentage of time spent in these 17 50-m depth bins, from the moment the whale started echolocating until the end of the record (S1 Table). To illustrate preferences in the depths at which buzzing or calling took place, we calculated ratios of probabilities for each depth bin: the percentage of buzzes or calls produced at a certain depth was divided by the percentage of time spent at that depth (Fig 5C and 5D). Ratios >1 (black fill) indicate more buzzes than expected based on the time spent there, while the opposite is true for ratios <1 (gray fill). Buzzes occurred much less than expected at 0–50 m, and at more than four times the expected rate in the range 350–650 m (Fig 5C).

Buzz duration was computed for a subsample of 193 buzzes (Table 3). For five out of six whales, median buzz length was in the range 2–3 s; Thora was the exception with a median buzz length of only 1.5 s and an interquartile range of less than one second. Unexpectedly long buzzes were also not unusual: amongst the randomly selected buzzes that were analyzed, four (2%) had durations exceeding 9 s, and a review of notes taken during manual analysis of the records showed that all whales but Mára produced buzzes more than 8 s long, and in a few cases 15–20 s in duration.

### Calls: numbers and depth distribution

A total of 1635 calls of various types were detected in the records, made by both tagged whales and other nearby whales (Fig 3, Table 3).

Peak frequencies of narwhal clicks are on average 69 kHz (± 14 kHz, [7]) and contain virtually no energy below 20 kHz. Because most calls made by narwhals are click-based, we did not expect to detect many calls made by whales other than the tag-bearer in the LF files. A comparison of the proportion of calls (from all sources) detected in the LF versus HF files for the three females with long records and alternating sampling confirmed this, with 20%, 14%, and 31% more calls than expected in the HF files of Freya, Thora, and Eistla, respectively.

The mean depth at which calls were detected was < 40 m in all cases and the median was less than 13 m (Table 3). More than 70% of calls were in the top 50 m, with only a few percent in other depth bins (Fig 5B). Because the whales also spent much of their time in the top 50 m (S1 Table), calls occurred at rates close to expected near the surface (Fig 5D). They occurred at about twice the expected rate at 50–100 m, and much less than expected at most other depths—for example, about 20x less than expected at 450–600 m (1 / 0.05 = 20). For all whales combined, an average of 54% of calls were produced in the top 7 m of the water column where the whales spent an average of 57% of their time (not shown). Probabilistically, near the surface is therefore where a narwhal is most likely to meet other narwhals and where social interactions are most likely to take place. This depth segregation between calls and buzzes is also visible in Fig 3.

### Probabilities of buzzing and calling as a function of *area*, *depth*, and *time-of-day*

Logistic regression modeling was used to assess the effects of geographic location, diving depth, and time-of-day on buzzing and calling rates for the five longer records. These analyses exclude the post-release silent period observed in all whales (Fig 3, Table 2) and begin as soon as a position was available, after the start of echolocation in each narwhal.

#### Effect of *area*

For buzzes, *area* was statistically significant for three of the four whales that visited more than one area: Thora, Eistla, and Balder (p < 0.0001). It was close to significant for Frida (p = 0.059), who spent nearly all her time in Gåsefjord, with only a short visit to Outer Gåsefjord—this aspect of her dataset decreased our ability to show a significant effect. For calls, *area* was statistically significant for all whales, with p < 0.0001 for Thora and Eistla, p = 0.0057 for Frida, and p = 0.044 for Balder.

For each whale, the odds ratios of producing buzzes or calls in the areas that the whales visited are summarized in Table 4. These odds ratios cannot be compared between whales, since they did not all visit the same areas and therefore do not have the same reference, but they remain useful for comparisons within each whale, and qualitative comparisons between whales. For example, both Thora and Eistla, who visited areas F, OG, and G, showed much lower buzzing rates in area F (Table 4): Thora, for example, buzzed about 14 times more in Outer Gåsefjord (OG) and 9 times more in Gåsefjord (G). Frida buzzed about twice as much in area G compared to OG, while Balder buzzed 15% less in area Ø than in area F. Table 4 also shows odds ratios that go in opposite directions for buzzes and calls (yellow versus blue backgrounds in Table 4). For example, Thora and Eistla were more likely to buzz in the Gåsefjord complex (OG and G) than in Fønfjord (F), but they were both more likely to call in the latter. This trend was present in all four whales.

**Table 4:** Odds ratios for buzzes and calls for the four whales that entered more than one area: Thora, Frida, Eistla, and Balder. Fønfjord (area F) is the reference for Thora, Eistla, and Balder, and Gåsefjord (area G) is the reference for Frida. Values indicating odds ratios > 1 are shown in **bold** on a yellow background, values indicating odds ratios < 1 are shown on a light blue background, and 95% confidence intervals on the odds ratios are shown in *italics*. NA = not applicable.

	Area:	Ø	F	OG	G
Thora	Buzzes	NA	1	**14.05**	**8.60**
	*95% C*.*I*.			*12*.*05–16*.*39*	*7*.*80–9*.*49*
	Calls	NA	1	0.38	0.95
	*95% C*.*I*.			*0*.*20–0*.*69*	*0*.*82–1*.*10*
Frida	Buzzes	NA	NA	0.56	1
	*95% C*.*I*.			*0*.*29–1*.*07*	
	Calls	NA	NA	**9.64**	1
	*95% C*.*I*.			*2*.*72–34*.*19*	
Eistla	Buzzes	NA	1	**1.80**	**2.04**
	*95% C*.*I*.			*1*.*60–2*.*02*	*1*.*81–2*.*30*
	Calls	NA	1	0.94	0.41
	*95% C*.*I*.			*0*.*76–1*.*18*	*0*.*33–0*.*52*
Balder	Buzzes	0.86	1	NA	NA
	*95% C*.*I*.	*0*.*81–0*.*92*			
	Calls	**1.40**	1	NA	NA
	*95% C*.*I*.	*1*.*01–1*.*95*			

**Fig 6:**
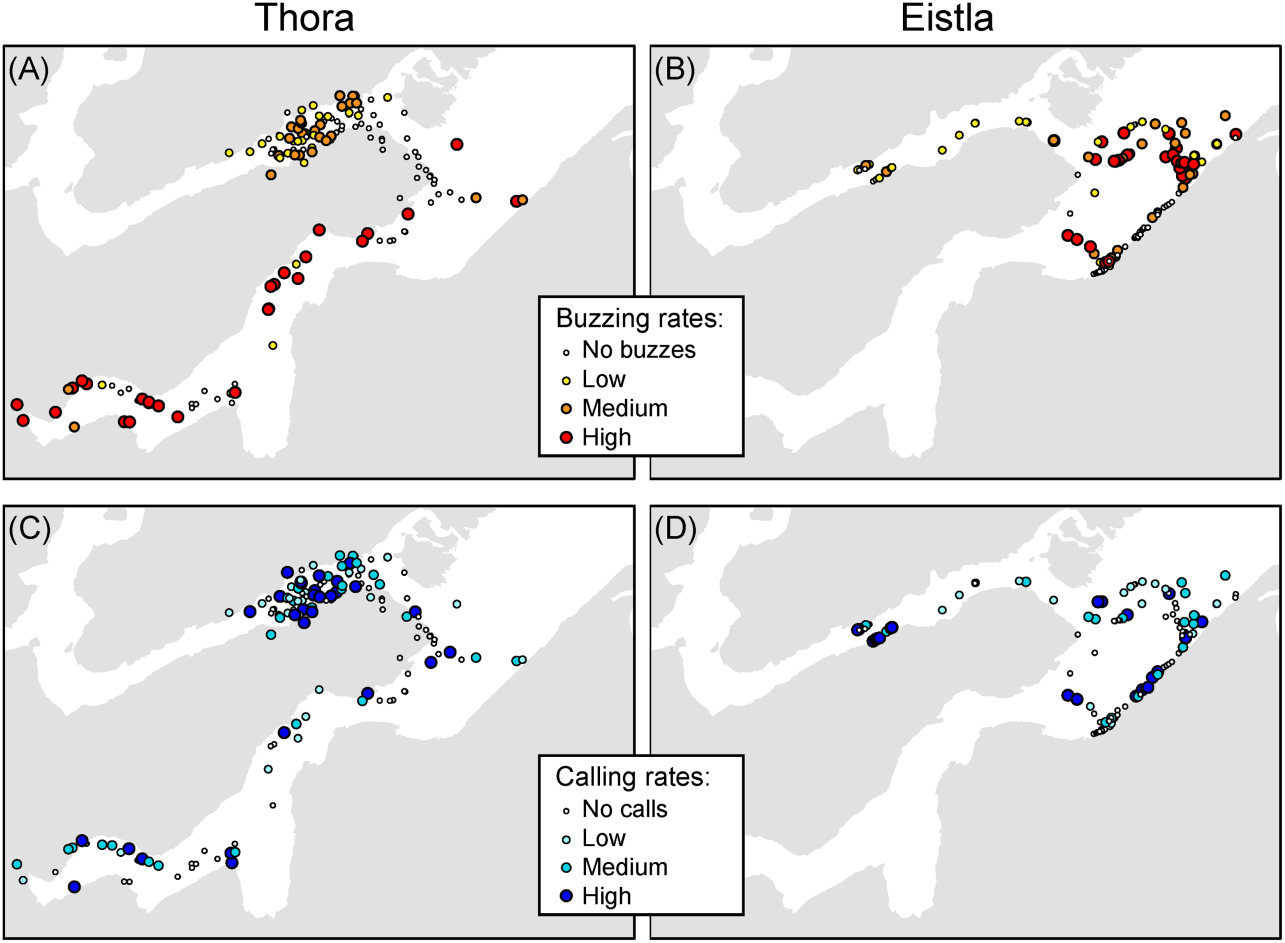
Buzzing and calling rates as a function of location for females Thora and Eistla. Locations devoid of buzzes or calls are shown as white circles. Locations with associated sound production are shown with increasingly darker and larger symbols, in a red color scheme for buzzes [panels (A) and (B)] and a blue color scheme for calls [panels (C) and (D)]. The three sound production categories were limited by the 33^rd^ and 66^th^ percentiles of the buzzing or calling rate for each female, with Low < 33^rd^ percentile, Medium > 33^rd^ and ≤ 66^th^ percentile, and High > 66^th^ percentile. These percentiles were as follows—Thora (left panels): 33^rd^ = 0.39 buzzes / min and 0.22 calls / min, 66^th^ = 0.96 buzzes / min and 0.64 calls / min; Eistla (right panels): 33^rd^ = 0.69 buzzes / min and 0.10 calls / min, 66^th^ = 1.01 buzzes / min and 0.33 calls / min.

Buzz and call densities as a function of specific satellite-track locations are shown for the two females with the densest positions in their satellite tracks, Thora and Eistla (Fig 6). Both carried Wildlife Computers SPLASH tags, but Eistla’s had the Fastloc^®^ GPS option, so the number of satellite hits and the accuracy of her localizations were much higher than Thora’s. For both females, the vast majority of positions with high buzzing rates (dark red dots) were in the Gåsefjord complex, whereas positions with high calling rates (dark blue dots) were more evenly spread in all areas visited.

#### Effect of *depth*

**Fig 7:**
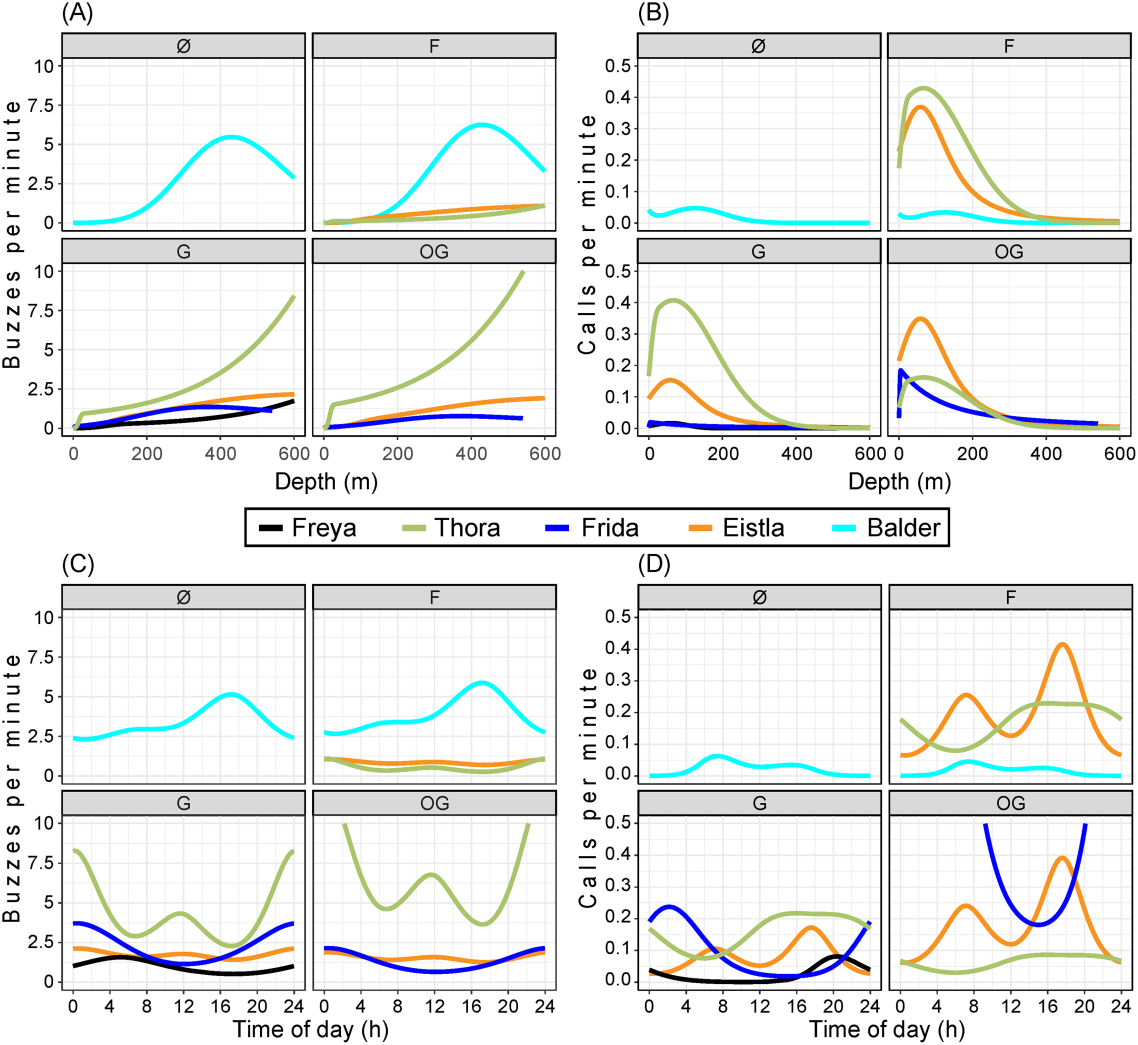
Logistic regression models. These express the relationship between *depth* [(A) and (B)] or *time-of-day* [(C) and (D)] and the occurrence of buzzes [(A) and (C)] or calls [(B) and (D)].

For buzzes, a non-linear effect of *depth* was statistically significant for all whales (p < 0.0001, Fig 7A). Buzzing rate increased with depth, at least down to 400 m, beyond which the paucity of data make interpretation difficult. Thora and Balder had the highest buzzing rates (as can also be seen in Fig 3), but in different areas, so the *whale* and *area* parameters are confounded.

For calls (Fig 7B), the non-linear effect of *depth* was statistically significant for all whales (p < 0.0001 for Thora, Frida, and Eistla, p = 0.019 for Freya, and p = 0.0002 for Balder). Fig 7B shows that the highest calling rates were at depths of less than 100 m, and that Thora and Eistla’s records had the highest calling rates.

#### Effect of *time-of-day*

For both buzzes (Fig 7C) and calls (Fig 7D), a non-linear effect of *time-of-day* was statistically significant for all whales (p < 0.0001). The four females produced most of their buzzes during the night, whereas Balder produced most of his buzzes in late afternoon (Fig 7C). There was a peak in calls in late afternoon or evening in the records of Eistla, Thora, and Freya (Fig 7D). Frida’s peak in calling was at night in the area (G) where she spent most of her time. Balder had the lowest overall rate of calls in his record, with a peak in the early morning.

## Discussion

To our knowledge, the acoustic behavior of narwhals in East Greenland has not been described before and the existing knowledge of narwhal vocalizations from West Greenland and Canada is fragmentary, based on short recordings with dipping hydrophones or a few hours of instrumentation with sound recorders. This paper describes the acoustic behavior of narwhals from Scoresby Sound over periods of up to seven continuous days, and thereby provides a glimpse into how these Arctic whales use sound in everyday life. We focus on the occurrence in time and space of three components of the whales’ acoustic repertoire: clicks, buzzes, and calls. We show a separation in time and space in the utilization of buzzes, used during feeding, versus calls, presumed to serve social communication. We also demonstrate a possible effect of tagging on the whales, which is important to take into account when interpreting tag data, particularly when records are of short duration.

### Clicking behavior

Narwhals spend on average about 27% of their time clicking (excluding the post-release silent period), with mean bout durations of 5.5–10 min. This is more than the echolocation duty cycle of about 17% for Blainville’s beaked whale (*Mesoplodon densirostris*) [30, 31], but likely less than the echolocation duty cycle in harbor porpoises (*Phocoena phocoena*), that have recently been shown to forage nearly continuously [32]. This finding may have implications for passive acoustic monitoring (PAM) of narwhals. Koblitz *et al*. [8] have reported an average -3 dB beamwidth for narwhals of 5°, the narrowest to date in an odontocete. If we combine the relatively low echolocation duty cycle, the narrow beamwidth, and the generally low densities of their populations, the probability of detecting a passing narwhal on a seafloor recorder is likely low. The high variability of the echolocation activity is an additional complicating factor when extrapolating acoustic recordings to population densities. This needs to be taken into account when using PAM for abundance estimates, such as has been done for harbor porpoises (*e*.*g*., http://www.sambah.org).

### Buzzing behavior

Buzzes are used during feeding by providing the whale with the fine-scale positional information needed over the last moments preceding prey capture [22, 23, 33, 34]. In this study, buzzes were sometimes produced near the surface—particularly for the female Frida—but visual inspection (Figs 3 and 5) as well as the results of the logistic regression models (Fig 7) show that most buzzing took place at depths below 250 m. Studies of stable isotope ratios [35] have determined that East Greenland narwhals primarily feed in the pelagic zone, a finding that is supported by their diving behavior [36]. Specifically, summer prey include mesopelagic species such as capelin (*Mallotus villosus*), Arctic cod (*Boreogadus saida*), polar cod (*Arctogadus glacialis*), squid (*Gonatus* sp.), and shrimp (*Pandalus* or *Crangon* sp.) [35, 37].

In addition to their depth stratification, buzzes were unevenly distributed in space, as buzzing rates were higher in the Gåsefjord complex (Figs 6 and 7, Table 4). We have no information on prey densities in Gåsefjord, but know from past satellite tagging that the fjord is one of two preferred areas used by the whales in summer [2]. It is also the calving ground of at least five glaciers, and there is evidence that glacial fronts are a preferred narwhal habitat [38]. In contrast, there are no calving glaciers in Fønfjord, between the field camp and the eastern tip of Gåseland (Fig 1), where buzzing rates were found to be lower for the two whales (Thora and Eistla) that buzzed in both areas.

Mean and maximum buzzing rates were 16–47 and 72–250 buzzes per hour, respectively (Table 3). Smaller odontocetes such as harbor porpoises are reported to produce up to ~500 buzzes / h [32], whereas sperm whales (*Physeter macrocephalus*) produce many fewer, on the order of 13 buzzes (creaks) / h, on average [22]. Cuvier’s beaked whales (*Ziphius cavirostris*), closer in size to narwhals, produce on average about 35 buzzes / h [39]. The narwhal buzzing rates reported here are therefore within the expected range.

Mean buzz duration was 1–3 s, again within expected range, but exceptionally long buzzes were also observed, lasting 10 s or more. In sperm whales, Miller *et al*. [22] has reported mean creak (buzz) durations of 8.7 s ± 7.6 s (S.D.), indicating the presence of creaks lasting 15–20 s.

*Time-of-day* had a statistically significant effect on the occurrence of buzzes, but the pattern was unclear, as peak buzzing activity occurred at different times in the females and the juvenile male.

### Calling behavior

In odontocete species that have been studied, non-feeding vocalizations are thought to serve communication during various social contexts, *e*.*g*., cohesion or recognition between individuals [40, 41]. Calls are also recorded in aggressive and agonistic interactions (*e*.*g*., [42]), and between mothers and calves [43].

Assigning calls to the tagged whale versus other nearby whales was sometimes problematic. To avoid this issue, we included all calls detected and used them as an indicator of ongoing communication. Most of our data (~75%) were obtained with a low sampling rate (~26 kHz), which is not ideal for recording odontocete click-based sounds except for those made by the tagged whale. We therefore surmise that only calling whales near the tagged whale, *i*.*e*., within a couple of body lengths, were audible in these recordings. Regardless, it is clear that calls (the majority of which are click-based) were mostly produced where the whales spent most of their time: in the top 50 m (58–86% of their time, S1 Table), or even in the top 7 m (44–70% of their time, not shown)—which is also therefore where they were most likely to encounter other narwhals. The different distribution in space and time of calls versus buzzes supports the notion that these sounds serve different purposes. Calling rates were always higher where buzzing rates were lower (Table 4). The effect of *subarea* (on calling rates) was statistically significant for all whales but the effect was weaker than for buzzes: locations with high calling rates in Thora’s and Eistla’s tracks (Fig 6C and 6D) are more scattered than the locations with high buzzing rates (Fig 6A and 6B).

The presence of other narwhals, as witnessed by the presence of their clicks, was not quantified, but the manual analysts noted that clicks from non-tagged whales were common in Thora and Eistla’s records but not in Freya’s (all three of these records included alternating LF / HF sampling). If calls serve social communication and social communication is favored by the presence of conspecifics, it is then not surprising that Thora and Eistla had the highest rates of calls in their records, 8.3 and 6.1 calls / h, respectively, compared to 1.2 for Freya (Table 3). Because of the difference in detectability of calls in the LF vs HF files, these values cannot be compared to those from the two records with continuous LF sampling (Frida and Balder). Nevertheless, Frida spent much more time near the surface than Balder (S1 Table, Fig 3), while the calling rate in her record was 2.5 times Balder’s (Table 3).

### Effect of tagging?

The data reported here suggest there is an effect of tagging. Whales abstained from clicking and buzzing for an average of 23 h after release (Table 2), but once echolocation began, click-free periods lasting more than a few hours were unusual (Fig 4B). Narwhals subjected to sound from ships cease vocalizing, a response thought to be similar to the whales’ response to killer whales [18]. Silence may therefore be a narwhal response to a stressor such as a real or perceived predator. In Shapiro [9], two narwhals were live-captured and instrumented with a satellite tag, a Crittercam, and an acoustic tag (DTAG). These records were short, lasting 2.5 h and 12.1 h, but neither contained regular echolocation clicks and buzzes (they did, however, include some “calls”, as defined in this paper).

A quantifiable effect of capture and instrumentation was recently obtained in a sister study on the same population of narwhals: Williams *et al*. [44] showed that the longer the handling time, the more drastic and long-lasting was the effect on diving bradycardia during the first few hours following release. In our study, the handling time was in the range 30–90 min, and depended on a variety of factors, including how far from camp the whale was caught and how many whales were caught at the same time. A simple linear regression revealed no relationship between handling time and the length of the post-tagging silence for the six whales included in this study, but that may simply indicate that other factors come into play.

An alternative or additional hypothesis to explain the long post-release silence is that Fønfjord—where all the whales initially headed following release—is not a preferred feeding area. Thora and Mára, who had the shortest post-release silent periods (9.4 and 9 h, respectively), both started echolocating and buzzing in Fønfjord, but (for Thora) at rates 9–14 times lower than later in Gåsefjord (Table 4). A similar observation can be made for Eistla, who returned to Fønfjord for the last 18.2 h of her record and was buzzing there, but at about half the rates she had in Gåsefjord and Outer Gåsefjord (Table 4). Balder, the male, headed east into Fønfjord after release, but then reversed direction and headed north into Øfjord (Figs 1 and 2), spending about half his record in each of these two areas. In contrast to the females, Balder had higher buzzing rates in Fønfjord than later in Øfjord, but only by an average of ~16% (Table 4).

### Data limitations

The population of East Greenland narwhals described in this study spends its summer traveling around the various fjords of the Scoresby Sound fjord system [2], one of the world’s largest (38,000 km^2^). The fjord system includes a number of different habitats that are used by the whales. The deployment durations achieved—4 to 7 days of continuous recording, depending on the sampling regimen chosen—are longer than most odontocete deployments to date (*e*.*g*., [9, 13, 45, 46]). Nevertheless, they still fall short of providing us with a complete picture of the summering ground habitat utilization, particularly for the areas that are known to be used by the population as a whole [2] but were not visited by the six Acousonde-carrying whales. Additionally, records are needed from adult males to examine possible sex differences in acoustic behavior.

## Conclusions

This study provides a basic description of the acoustic behavior of East Greenland narwhals, which will serve as a point of comparison in ongoing assessments of the effects of airgun pulses on these whales. We also believe the records presented here are the longest obtained to date on free-ranging odontocetes. Such multi-day records are vital for describing aspects of a species’ life history, foraging ecology, and habitat utilization, and therefore also for assessing the whales’ resilience to changes in their environment, such as those that may be brought on by changes in climate.

This study shows that narwhals can be tagged with archival acoustic tags for periods of up to a week. The tags, such as the Acousondes used in this study, can successfully be retrieved even in remote locations such as East Greenland if they are also fitted with a satellite tag. Furthermore, basic information on narwhal echolocation behavior can be obtained while using surprisingly low sampling rates (25.8 kHz), but only if suction cups provide a tight connection to the animal’s body. This is important because current battery and storage limitations require low sampling rates if continuous recordings are needed over periods of many days. The integration of data from high-accuracy satellite tags, such as the SPLASH tag with the Fastloc^®^ GPS option, with information from multi-sensor tags such as the Acousonde should lead to novel insights about area-use patterns, feeding behavior, and other aspects of marine mammal ecology.

## Supporting information

S1 Audio Distinguishing clicks made by tag-bearer vs other narwhals. (WAV)

S2 Audio Distinguishing clicks made by tag-bearer vs other narwhals. (WAV)

S3 Audio Distinguishing clicks made by tag-bearer vs other narwhals. (WAV)

S4 Audio Echolocation and buzzing, high sampling rate. (WAV)

S5 Audio Echolocation and buzzing, low sampling rate. (WAV)

S6 Audio Echolocation and buzzing, low sampling rate and high-pass filter. (WAV)

S7 Audio Calls by several narwhals in close proximity. (WAV)

S1 Fig Clicking and buzzing examples from files sampled at high and low sampling rates. (PDF)

S2 Fig Difference in the frequency composition of clicks produced by narwhals carrying the tag versus other narwhals. (PDF)

S1 File Information on the contents of the audio files S1 to S7 Audio. (PDF)

S1 Table Percentage time spent in 17 50-m depth bins, for all whales. (PDF)
