# Supplementary material for: Spatial and temporal patterns of sound production in East Greenland narwhals
Source: PLoS One. 2018 Jun 13;13(6):e0198295. doi: 10.1371/journal.pone.0198295 (PMC5999075; doi:10.1371/journal.pone.0198295)

S1 Fig. Clicking and buzzing examples from files sampled at high and low sampling rates. (A) Sample from a high-frequency file (154,868 Hz sampling rate). (B) Sample from a low-frequency file (25,811 Hz sampling rate). (C) Same sample as (B), but high-pass filtered at 1.5 kHz. Arrows point to (terminal) buzzes. All samples shown are from Freya's record.

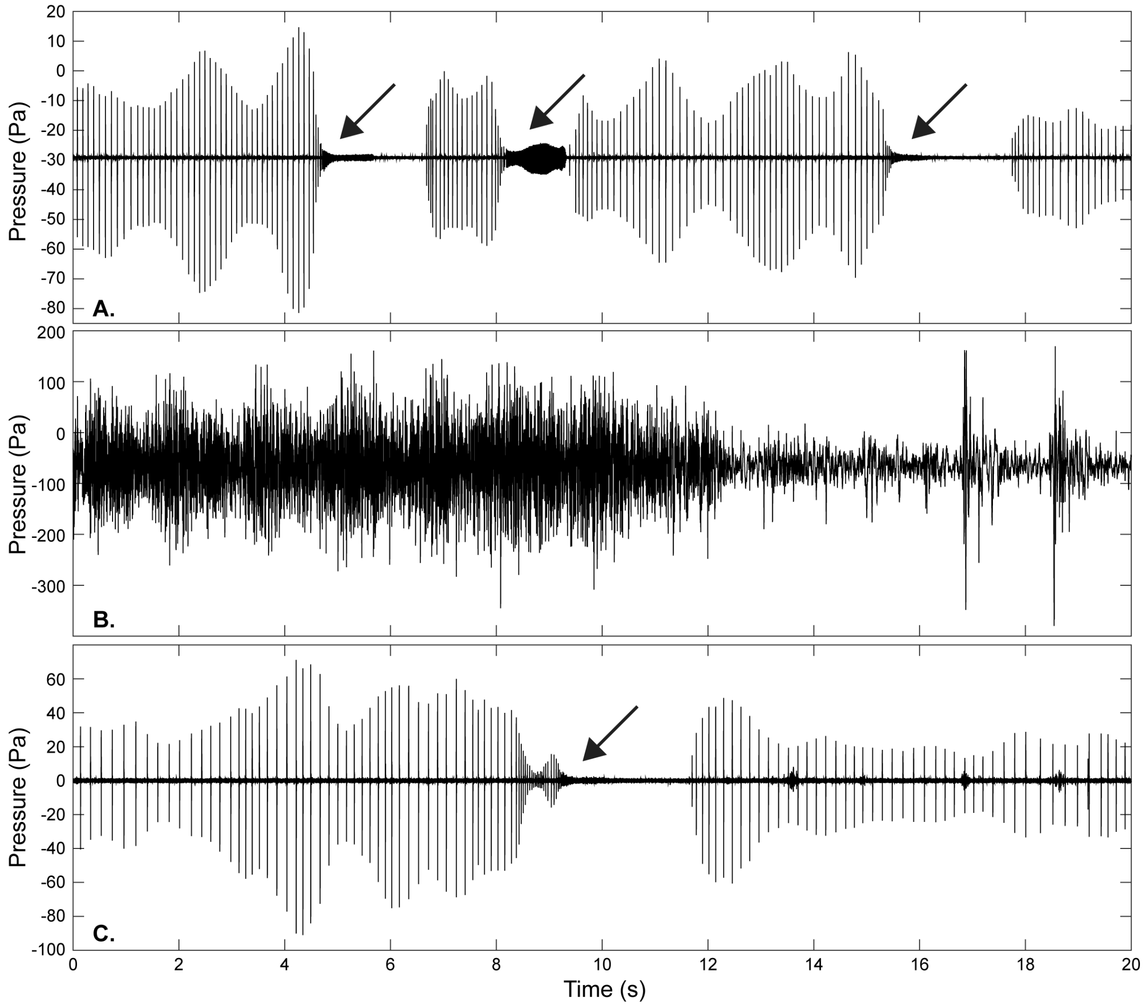

Supplement: S1 Fig — (PDF) [file pone.0198295.s008.pdf]
