# Supplementary material for: Spatial and temporal patterns of sound production in East Greenland narwhals
Source: PLoS One. 2018 Jun 13;13(6):e0198295. doi: 10.1371/journal.pone.0198295 (PMC5999075; doi:10.1371/journal.pone.0198295)
Supplement: S2 Fig — (PDF) [file pone.0198295.s009.pdf]

S2 Fig. Difference in the frequency composition of clicks produced by narwhals carrying the tag versus other narwhals. (A) Spectral density levels were computed for four clicks from each of the four whales whose records included high-frequency sampling. The levels were then normalized and a mean level (thick black line) was calculated in the linear domain. Fast Fourier Transform Length (NFFT) = 75, Hann window (Matlab function hanning()). (B) Three of the four records mentioned in (A) (all but Mára's short record) included clicks from narwhals other than that carrying the tag. Spectral density levels of eight clicks from each record (in three different years) were analyzed as in (A). NFFT = 75, Hann window (Matlab function hanning()).

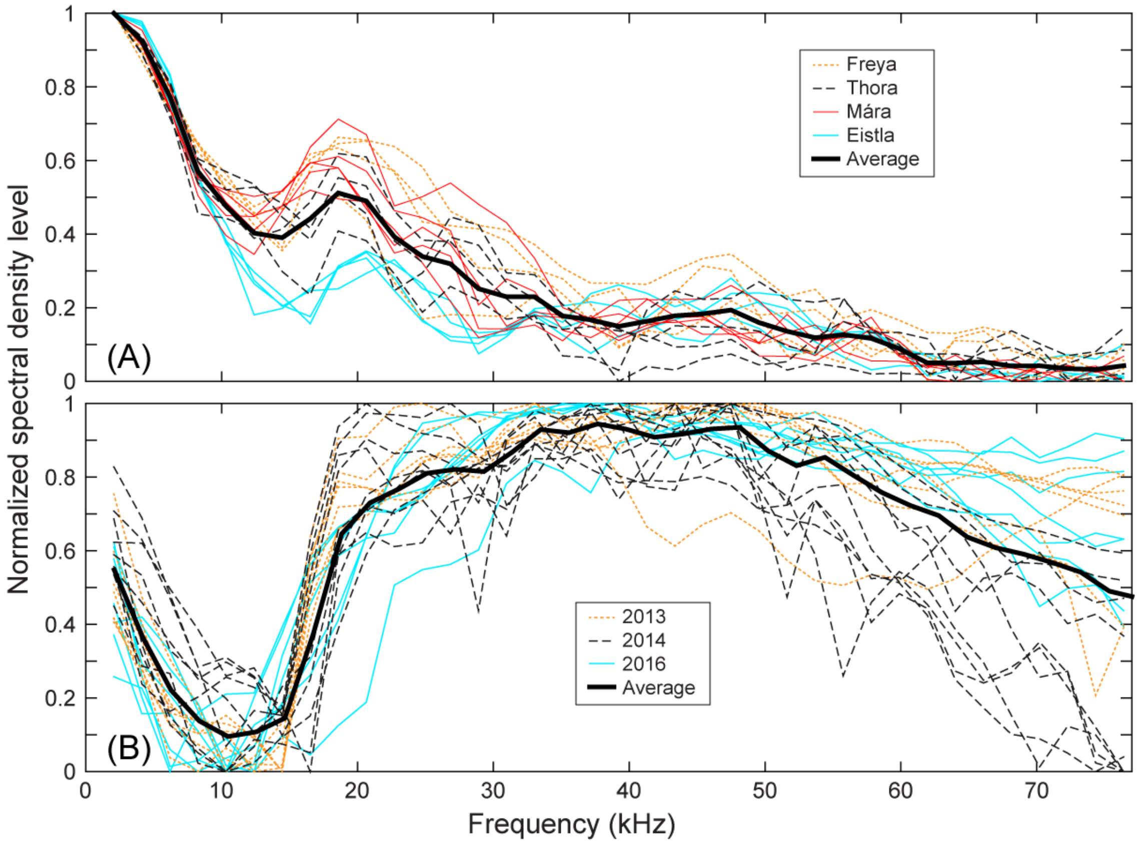

Note: the Acousonde has a 40 kHz cut-off frequency for the anti-alias filter, above which received levels decrease gently with increasing frequency. No adjustments were made in these figures for that anti-alias filter.
