# Supplementary material for: Spatial and temporal patterns of sound production in East Greenland narwhals
Source: PLoS One. 2018 Jun 13;13(6):e0198295. doi: 10.1371/journal.pone.0198295 (PMC5999075; doi:10.1371/journal.pone.0198295)
Supplement: S1 File — (PDF) [file pone.0198295.s010.pdf]

S1 File. Information on the contents of the wav files S1 Audio to S7 Audio. When listening to the files, the analysts always played the HF files at half speed, which allowed them to hear sounds with frequencies over 20 kHz. To provide the same experience here, the HF sound clips (S1 Audio, S2 Audio, S3 Audio, and S4 Audio) have been saved at half the original sampling rate (0.5 x 154,868 = 77,434 Hz). As a result, these sound clips have twice the length of the original versions.

S1 Audio. Distinguishing clicks made by tag-bearer vs other narwhals. Thora's record, 13 Aug 2014 at 21:55:55 (GMT), original sampling rate 154,868 Hz, diving depth ~38 m. Another narwhal can be heard clicking in the background while Thora makes a short series of clicks 4–6 s into the file (2–3 s into the original recording).

S2 Audio. Distinguishing clicks made by tag-bearer vs other narwhals. Eistla's record, 25 Aug 2016 at 16:09:25 (GMT), original sampling rate 154,868 Hz, diving depth ~250 m. Another narwhal can be heard clicking during Eistla's break in echolocation, 4–7 s into the file.

S3 Audio. Distinguishing clicks made by tag-bearer vs other narwhals. Eistla's record, 27 Aug 2016 at 17:40:18 (GMT), original sampling rate 154,868 Hz, diving depth 173–164 m (during ascent). Other narwhals can be heard clicking throughout the file; Eistla produces 8 loud and 2 weaker clicks 8–11 s into the file. This example demonstrates that amplitude alone cannot distinguish clicks made by the tag-bearer vs other narwhals.

S4 Audio. Echolocation and buzzing, high sampling rate. Thora's record, 14 Aug 2014 at 23:06:11 (GMT), original sampling rate 154,868 Hz, diving depth ~311 m. 5 s into the file, Thora starts a buzz lasting about 4 s (8 s in the file).

S5 Audio. Echolocation and buzzing, low sampling rate. Mára's record, 12 Aug 2014 at 1:39:39 (GMT), sampling rate 25,811 Hz, diving depth 313–337 m. 3.95 s into the file, Mára starts a buzz lasting about 2.1 s.

S6 Audio. Echolocation and buzzing, low sampling rate and high-pass filter. Same as previous file, but this file has been high-pass filtered at 1.5 kHz to minimize flow noise and emphasize echolocation clicks.

S7 Audio. Calls by several narwhals in close proximity. Thora's record, 14 Aug 2014 10:58:16 (GMT), sampling rate 25,811 Hz, data high-pass filtered at 1.5 kHz, diving depth ~16 m. In social situations with several nearby whales, determining the source of a call is not possible when sampling on the low-frequency channel.
