# Supplementary material for: Spatial and temporal patterns of sound production in East Greenland narwhals
Source: PLoS One. 2018 Jun 13;13(6):e0198295. doi: 10.1371/journal.pone.0198295 (PMC5999075; doi:10.1371/journal.pone.0198295)
Supplement: S1 Table — (PDF) [file pone.0198295.s011.pdf]

S1 Table. Percentage time spent in 17 50-m depth bins, for all whales. Values were calculated from the moment the whales started echolocating (see Table 2) until the end of their record (see Table 1).

Depth bin (m)	Freya	Mára	Thora	Frida	Eistla	Balder
0-50	61.67	30.10	69.95	85.84	57.62	65.98
50-100	5.90	7.27	4.72	3.67	4.03	7.34
100-150	4.79	8.59	2.89	3.03	3.17	4.15
150-200	4.82	10.22	2.16	2.22	3.04	2.78
200-250	4.02	8.87	2.09	1.68	3.25	3.42
250-300	4.38	5.89	2.80	1.08	3.82	3.10
300-350	4.66	9.64	3.96	0.98	4.34	3.05
350-400	3.50	8.10	3.09	0.84	5.29	3.23
400-450	2.49	2.89	2.21	0.47	3.95	2.77
450-500	1.66	2.01	2.56	0.14	4.45	2.12
500-550	1.05	4.46	1.90	0.05	3.06	1.23
550-600	0.67	1.96	0.92	0.00	1.31	0.52
600-650	0.35	0.00	0.33	0.00	0.94	0.17
650-700	0.03	0.00	0.26	0.00	0.76	0.02
700-750	0.00	0.00	0.08	0.00	0.71	0.03
750-800	0.00	0.00	0.06	0.00	0.19	0.05
800-850	0.00	0.00	0.02	0.00	0.06	0.04
